# Structural insights into the function of the core-circadian factor TIMING OF CAB2 EXPRESSION 1 (TOC1)

**DOI:** 10.1186/1740-3391-6-3

**Published:** 2008-02-25

**Authors:** Elsebeth Kolmos, Heiko Schoof, Michael Plümer, Seth J Davis

**Affiliations:** 1 Max Planck Institute for Plant Breeding Research, Carl-von-Linné-Weg 10, D-50829 Cologne, Germany

## Abstract

**Background:**

The plant circadian clock has at its core a feedback loop that includes TIMING OF CAB2 EXPRESSION 1 (TOC1). This protein has an as of yet unknown biochemical activity. It has been noted that the extreme amino-terminus of this protein is distantly related in sequence to response regulators (RR), and thus TOC1 is a member of the so-called pseudo response regulator (PRR) family. As well, the extreme carboxy-terminus has a small sequence stretch related to the other PRRs and CONSTANS (CO)-like proteins, and this peptide stretch has been termed the CCT (for CONSTANS, CONSTANS-LIKE, TOC1) domain.

**Methods:**

To extend further our understanding of the TOC1 protein, we performed a ROSETTA structural prediction on TOC1 orthologues from four plant species. Phylogenetic interpretations assisted in model construction.

**Results:**

From our models, we suggest that TOC1 is a three-domain protein: TOC1 has an amino-terminal signaling-domain related to response receivers, a carboxy-terminal domain that could participate both in metal binding and in transcriptional regulation, and a linker domain that connects the two.

**Conclusion:**

The models we present should prove useful in future hypothesis-driven biochemical analyses to test the predictions that TOC1 is a multi-domain signaling component of the plant circadian clock.

## Background

Circadian clocks are prevalent timing mechanisms used to predict the daily changes present in the 24-h day-night cycle. In plants, this clock regulates several developmental and metabolic processes. Dominant outputs include the oscillation of free-cytosolic calcium (Ca^2+^) [1], which are generated from cADPR-derived signals [2], and the rhythmic accumulation of around 10% of all transcripts [2-6]. In particular, transcription factors are over-represented as cycling gene products [3,7]. In this way, the circadian timer drives numerous molecular outputs in the establishment of fitness in physiological processes and developmental timing. This fitness benefit has been confirmed [8]. The current aims on studies of the mechanism of the plant clock are to define the factors that contribute to rhythm-generating properties of the oscillator.

Molecular-genetic analyses have lead to a framework understanding of the core elements that make up the circadian clock. Mutants of *Arabidopsis thaliana *that are clock defective have been used to identify loci critical for normal rhythmicity. *TIMING OF CAB2 EXPRESSION 1 *(*TOC1*) was the first such locus identified [9], and *TOC1 *continues to be placed central within the clock mechanism [10-14]. Extending from these studies, many clock genes are reciprocally regulated, and thus the transcriptional components that drive the clock are themselves clock controlled. Using this analytical approach, with a focus on molecular-expression analyses in clock mutants, the first model that partially explained mutant behavior was described [15]. In this model, *TOC1 *serves as an evening-expressed positive factor that regulates the morning expression of *CIRCADIAN CLOCK ASSOCIATED 1 *(*CCA1*) and *LATE ELONGATED HYPOCOTYL *(*LHY*) [15-18]. The central role of *TOC1 *has been genetically confirmed [10,11], but although TOC1 is unquestionably important for the circadian clock, lack of functional biochemical understanding has hampered characterization of its functional role within the oscillator.

Multiple regions of the *TOC1 *coding region are susceptible to mutagenesis. Weak mutations, such as the *toc1-1 *and *toc1-3 *alleles (both A562V changes within the carboxy-terminal portion) result in clock-specific defects. As well, missense mutations in the amino terminus of TOC1 have been isolated from direct circadian screens [*toc1-5 *(P124S); *toc1-8 *(P96L)] [19,20]. In contrast, null mutants, such as *toc1-2 *(splice site mutation that leads to N-terminal 1–59 aa fragment) and *toc1-21 *(a null allele derived from a T-DNA insertion), have defects both in circadian properties and in light signaling [10,21,22]. Thus, TOC1 can have multiple physiological roles that can be genetically separated.

To date, the only defined activity within any region of the TOC1 polypeptide is a nuclear-trafficking signal established by the CCT motif (for CONSTANS, CONSTANS-LIKE, TOC1) in the carboxy-terminus [22,23]. It has been previously noted that the amino-terminal domain resembles in its primary structure sequence conservation with bacterial-type response regulators (RR) [23]. This domain in TOC1 thus places it as a founding member of the pseudo-response-regulator (PRR) protein family. The function of the pseudo-receiver domain is unknown, because results of *in vitro *experiments confirm that the PRR domain does not undergo phosphorylation, as suspected, due to a lack of a conserved Asp within the response-receiver [23]. One collective interpretation proposed here, which incorporates these diverse experiments, is that TOC1 is a multi-domain protein. TOC1 thus integrates signal inputs that bridge multiple physiological responses [24]. That weak mutations can be uncovered which only display a subset of phenotypes [15,22] support our hypothesis of multiple signaling functions of TOC1.

Diurnal calcium (Ca^2+^) rhythms are evident in the plant cell. The daily rise and fall of free-cytosolic calcium has been proposed to encode a photoperiodic signal [25-27]. The signaling nature of the encoded rhythmic Ca^2+ ^is an active area of investigation [25,27,28], and the receptor for this Ca^2+^-derived signal is as of yet unknown. One point of note is that the phase of calcium increase is coincident with that seen with TOC1 protein levels, as both occur around dusk [26,29]. Therefore, it would be of interest to define whether evening factors such as TOC1 comprise part of a decoding mechanism of the Ca^2+ ^signal.

In this work we used modeling and phylogenetic approaches to further dissect the TOC1 protein sequence. Several TOC1 polypeptides were detected in sequence databases. These TOC1 proteins appear to contain three distinct modules. Computational approaches using the ROSETTA suite of programs lead to the development of structural models of the TOC1 modules. One interpretation of these structures is the implication that TOC1 functions as a signaling protein that in part works to process calcium information in the induction of transcriptional responses.

## Methods

### Defining TOC1 orthologous sequences

To assess putative structures of TOC1, as it relates to differences with the PRR related sequences, we searched public sequence databases for genes that encode full-length proteins. The following Genbank accessions were used: *At*TOC1 (NM_125531), *At*PRR3 (NM_125403), *At*PRR5 (NM_122355), *At*PRR7 (NM_120359), *At*PRR9 (NM_201974), *Os*TOC1 (AB189038), *Os*PRR37 (AB189039), *Os*PRR73 (AB189040), *Os*PRR95 (AB189041), *Os*PRR59 (ABA91559), *Cs*TOC1 (AY611028), *Lj*TOC1 (AP004931), *Mc*TOC1 (AY371288), *Pt*TOC1 (NW_001492741), and *Vv*TOC1 (CAO64513)

For phylogenetic confirmation of TOC1 sequence identification, polypeptides where clustered using CLUSTALW [30], and this was used to generate a tree using UPGMA, where CLC FREE WORKBENCH (CLC bio, Aarhus, Denmark) facilitated these efforts.

### Modeling and model comparisons

The ROSETTA software suite was generously supplied by the Baker Laboratory (University of Washington, Seattle, USA) and it was used to model the three modules of four selected TOC1 polypeptides; each were modeled 500 times. These models were clustered, and up to 10 consensus structures for all four given domains were compared by SARF2 [31]. From this, those structures most related were taken forward for comparisons. These 12 structures are available as supplemental files in PDB format (see Additional files 1, 2, 3, 4, 5, 6, 7, 8, 9, 10, 11, 12). The three-dimensional domains were aligned and visually presented using MACPYMOL 0.99 (DeLano Scientific LLC, Palo Alto, USA). Related structures were found with SSM [32]. Calcium was fit using the GG method [33]. The bacterial response regulators were CheY (PDB code 1E6K) and SPO0F (PDB code 1SRR). A PDF file of the CCT domain of CONSTANS was provided by Dr. Coupland.

## Results and discussion

### Phylogenetics

We sought to detect TOC1-related sequences from various plants as a phylogenetic starting tool for structural predictions. For this, *At*TOC1 [22] and *Os*TOC1 [34] were used to search genome-sequence databases. Full-length predicted proteins were found for *Castanea sativa*, *Lotus japonicus*, and *Mesembryanthemum crystallinum*, and more recently, *Vitis vinifera *and *Populus trichocarpa*. These full-length sequences were chosen as they were reported to exhibit the architecture typical to TOC1, as was defined previously by the Mizuno group [35]. Out-group sequences were the paralogues of the PRR family, which are PRR3/5/7/9 from Arabidopsis, and from rice (*Oryza sativa*), *Os*PRR37 and *Os*PRR73, *Os*PRR59 and *Os*PRR95 (rice PRR5 and PRR9 have not yet been phylogenetically resolved from each other, nor have rice PRR3 and PRR7) [23,34].

**Figure 1:**
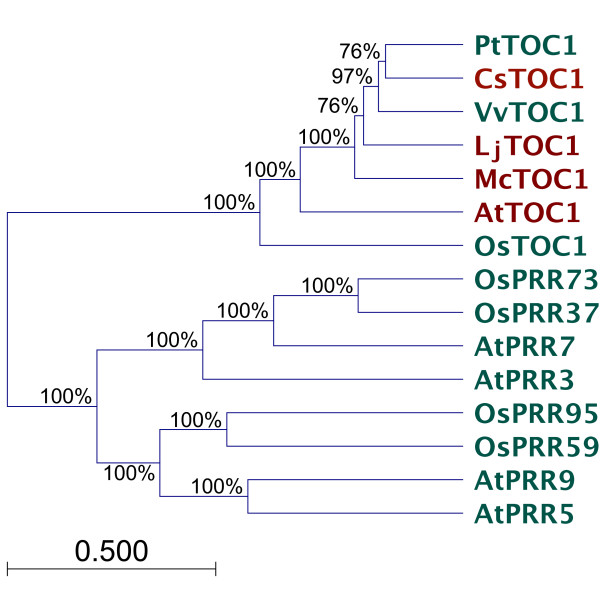
**TOC1 and PRR phylogeny**. UPGMA phylogenetic tree of TOC1/PRR proteins. The groupings are strongly supported, as indicated by high bootstrap values (>70%). The scale bar represents 0.05 estimated amino-acid change per sequence position. Sequences in red were selected for further analysis in this study. Pt, *Populus trichocarpa*; Cs, *Castanea sativa*; At, *Arabidopsis thaliana*; Vv, *Vitis vinifera *Lj, *Lotus japonicus*; Mc, *Mesembryanthemum crystallinum*; Os, *Oryza sativa*. Sequence origin can be found in the Methods section.

We generated a phylogenetic tree using UNWEIGHTED PAIR GROUP METHOD WITH ARITHMETIC MEAN (UPGMA) clustering and a bootstrap replicate number of 10,000 to confirm that the encoded proteins isolated from databases were the orthologues of TOC1 and paralogous to the other PRRs. As can be seen in Figure 1, the sequences *Cs*TOC1, *Lj*TOC1, *Mc*TOC1, *Pt*TOC1, and *Vv*TOC1 all clustered with the rice and Arabidopsis TOC1 proteins, as expected. Because it would have been computationally too intense to model all TOC1 polypeptides, a selection of four was taken forward. These representatives were *At*TOC1, *Cs*TOC1, *Lj*TOC1, and *Mc*TOC1; noted in red in Figure 1. We further reasoned that the use of four structural models of orthologous sequences would provide a template to assign the relatedness of any one given structure.

### Model predictions of TOC1

**Figure 2:**
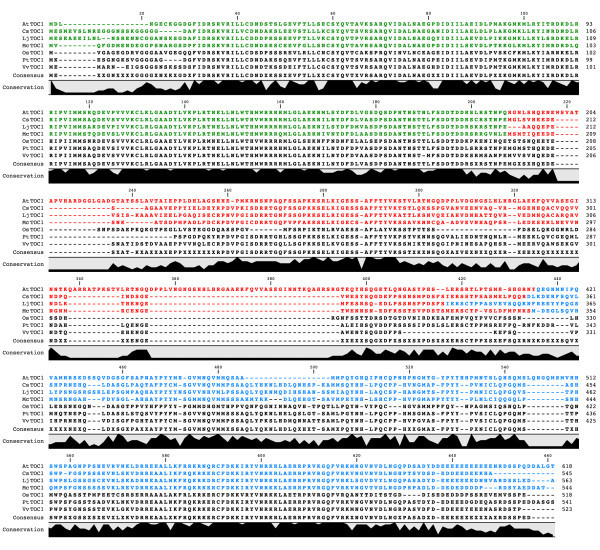
**Global alignment of selected TOC1 sequences**. ClustalW multiple alignment of TOC1 amino-acid sequences chosen based on the phylogenetic analysis in Figure 1. The three colors (green, red and blue) represent the modular domains for the four TOC1 sequences that were selected for further analysis by defining regions in sequence that move from conservation to non-conservation. The conservation block highlights the percentage identity of amino-acids in the lineup. Note that for module I and module III, there is far more identity than in module II. Abbreviations refer to: At, *Arabidopsis thaliana*; Cs, *Castanea sativa*; Lj, *Lotus japonicus*; Mc, *Mesembryanthemum crystallinum*; Os, *Oryza sativa*; Pt, *Populus trichocarpa*; Vv, *Vitis vinifera*

We sought to infer tertiary structure of TOC1 using *ab initio *approaches through the ROSETTA software suite. This suite provides one strategy towards understanding potential folds of a target protein starting simply with the primary amino-acid sequence [36,37]. The TOC1 sequences are computationally too large for complete structural solution by ROSETTA as a single polypeptide chain [36], thus putative folding modules within the sequences were required to be defined. Here, a folding module is defined as a unit within the polypeptide required for a given biochemical activity. To define modules, the full set of above defined TOC1 proteins were aligned (Figure 2) and the transition areas in the lineup where sequence conservation moves to non-conservation was noted (color points to these transitions is indicated in Figure 2). These informatic "cut sites" are estimates of folding modules [38]. By this approach, TOC1 could be dissected into three domain modules (Figure 2). With respect to the *At*TOC1 protein, these modules were from amino-acid positions 1–189, 190–412, and 413–618, respectively. As four TOC1 sequences were to be applied to ROSETTA, with three modules each, we therefore proceeded with predicting structures for twelve separate polypeptide domains.

Each module was edited from the four respective full-length proteins and modeled separately. A family of 500 models of each module was generated and these were clustered based on the free-energy landscape within these, leading to groups of up to 10 related structural families. In these clusters, the structure centered within a given cluster was selected as the representative of said cluster. For this, ROSETTA determines an all-atom energy axis and plots this against an axis of the ROOT MEAN SQUARE DEVIATION (RMSD) of the resultant structures [36]. From there, each of the related four proteins of each module was processed on SPATIAL ARRANGEMENT OF BACKBONE FRAGMENTS 2 (SARF2) [31] as an approach to define those structures within clusters that most resembled likeness to orthologous structural domains. We note that SARF2 was developed as a clustering approach that detects ensembles of secondary-structure elements that form similar spatial arrangements, whilst accepting different possible topological connections [31]. With this approach, we found within the identified structural clusters the subclade with the best statistical fit, as assessed by RMSD, for a given structural module. Combining the representative clustering of ROSETTA to the relatedness clusters of SARF2 lead to one choice for each module within a given sequence. The resultant structures from this method were thus selected as the most representative of a given structural protein module. What follows is a description of each model and our discussion of the implications for that particular module.

#### Models of module I

**Figure 3:**
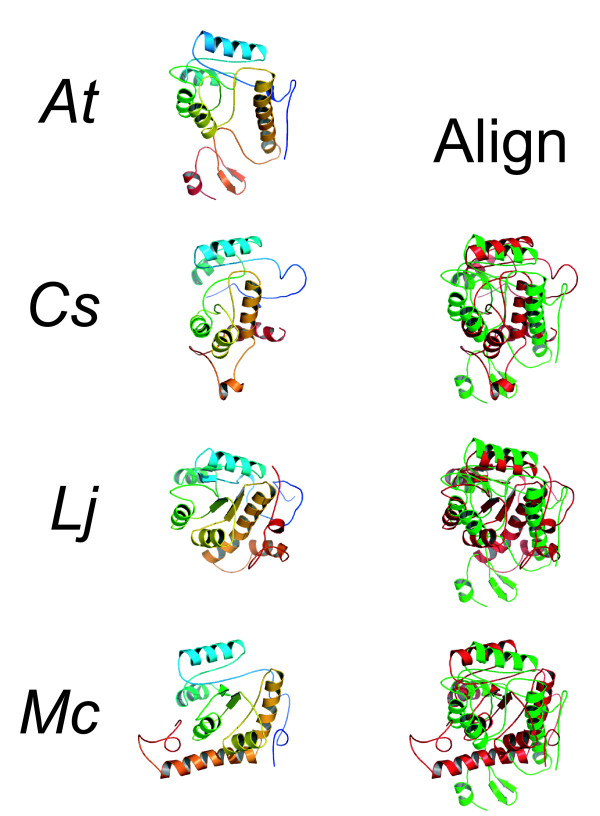
**Models of module I**. Structural models of module I (left) and aligned with the Arabidopsis domain I (right). For the images at the left, the colors from blue to red represent sequence length from an amino- to carboxy-terminal direction. For the aligned figures at the right, the Arabidopsis module I is colored green in contrast to a red color for the compared alignment.

**Figure 4:**
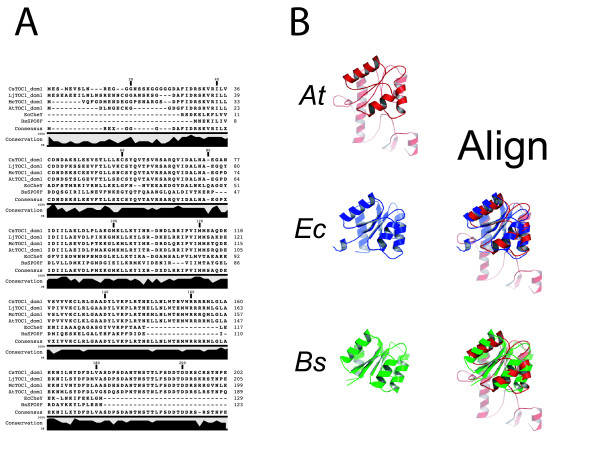
**Comparison of module I to response regulators from bacteria**. (A) Multiple alignment of module I from plants and response regulators from bacteria. Ec, *Escherichia coli *CheY; Bs, *Bacillus subtilis *SPO0F. The lineup is as described in Figure 2. (B) Structures of the Arabidopsis model for module I and published structures for two response regulators (left) and aligned with to Arabidopsis module I (right). Coloration is as shown.

We first generated protein models for the amino-terminal third of the TOC1 polypeptides (Table 1, Figure 3). These models were highly related in structure to each other (Figure 3). Using a query of the generated structures against all known protein structures at the Protein Data Bank, *via *the use of the software SECONDARY STRUCTURE MATCHING (SSM) [32], we found that all models were predicted to fold similarly to bacterial RR proteins (data not shown; see below for discussion and Figure 4 for representative example) [39,40]. Generally, all module I structures have a core of five alpha helices interdigited with alternating beta sheets. This resembles the canonical fold of all RR structures. As well, an alpha-helical tail extends from the RR-like portion of the structure.

**Table 1:** The table summarizes the number of selected cluster-center modules chosen from the starting point of 500 generated ROSETTA structures (see Methods).

	Module I	Module II	Module III
*At*TOC1	3	10	10
*Cs*TOC1	4	8	3
*Lj*TOC1	5	9	9
*Mc*TOC1	3	10	8

The mutations *toc1-5 *(P124S) and *toc1-8 *(P96L) lay within module I, and the *At*TOC1 structure allows examination of where this mutation would perturb function. Amino acid 96 is in a predicted beta sheet that bridges helix three and four. This proline mutation might disrupt folding activity as a structural mutation. Amino-acid position 124 is in a loop between helix four and five. Whilst this could be a structural mutation, this position does not lie within an obvious folding pattern. The P124S mutation might affect TOC1 binding to a putative associated molecule (see "additional files" to retrieve the PDB files to expand a view on these, and all other, structures).

The RR class of proteins mediates phospho-relay signaling in bacteria and plants [41,42]. That the amino terminus of TOC1 was predicted to fold like an RR is not a surprise, as the primary sequence of this domain is detected by BASIC LOCAL ALIGNMENT SEARCH TOOL (BLAST) [43] as resembling an RR. We found that a superimposition of the Arabidopsis model on two *bona fide *RR crystal structures (*Escherichia coli *CheY and *Bacillus subtilis *SPO0F [44-46]) reveals an excellent structural fit (Figure 4). We note that there is an amino- and carboxy-terminal extension of the first domain of TOC1 relative to the two bacterial proteins tested.

A structure resembling an RR implicates an origin of function for the amino-terminal module of TOC1. This further supports the phylogeny relations of the amino-terminal module of PRR to genuine RRs [40]. In each of the four TOC1 modules, an Ala is present at what is the Asp site of phosphorylation in a *bona fide *RR. In the illustrated models for module I (Figure 3), this Ala is predicted to be within the center of the five alpha-helical borders. This is all consistent with the previous hypothesis that TOC1 is not a substrate of a histidine kinase [22]. As the structures generated all resemble an RR (Figures 3 and 4; and data not shown), we conclude that these models are likely to resemble the "true" fold of this domain module.

What could be the function of an RR-domain-type fold within module I of TOC1, particularly as it appears incapable of functioning as a true RR? Several possibilities exist. For one, this domain could be a protein-binding site incorporating, *via *a scaffold function, the activities of other clock proteins, as for example, transcription factors. Specifically, TOC1 is known to bind members of the bHLH transcription factor family (*e.g*. PIL1, PIF3, PIF4, PIL6) [47,48]. However, in these studies, the RR domain was shown not to be required for binding of PIF4 or PIL6 [49]. PRR proteins can also form dimers, and in case of TOC1 binding to PRR9, PRR9 was found to interact with TOC1 through the RR domain [49]. Furthermore, an important role of the RR domain in protein-protein interaction was found for PRR3 when defined as a substrate of the kinase WNK1 [50,51]. In addition, it is not yet established if the ZEITLUPE (ZTL) or the PRR3 binding sites associate with the RR domain [13,29]; both ZTL and PRR3 are confirmed protein interactors to TOC1. It is also plausible that the RR-type domain/module could be a redox-responsive site, as was hypothesized by the work of the Golden group [52,53]. What appears clear is that identification of interacting molecules to the amino-terminal module will likely define a biochemical function.

#### Models of module II

**Figure 5:**
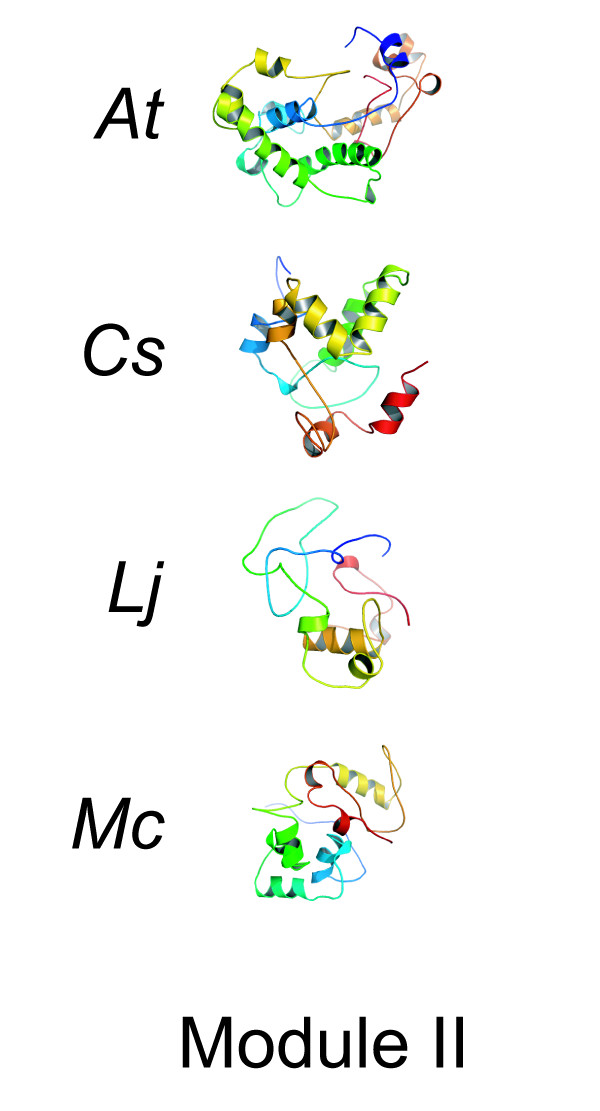
**Models of module II**. Structural models of module II. The colors from blue to red represent sequence length from the amino- to carboxy-terminal direction.

Our next efforts were to model the middle third of the four TOC1 modules. These predictions were found to be structurally unrelated to each other (Figure 5, Table 1). This is of interest as the primary amino-acid composition of the middle third is the most distinct (Figure 2). We note that this is true for the other PRR proteins as well [54]. The lack of a consensus structure within the middle third of the polypeptide (Figure 5) prohibits us from making any structural conclusions. As well, this module lacks relations to other structural features bioinformatically characterized. One small amino-acid stretch is conserved in the second module; respective to *At*TOC1 module II, the sequence is KKSRLKIGESSAFFTYVKST. Examination of this stretch within module II of the four predicted structures revealed no fold consensus. It is thus difficult for us to predict the reliability of the presented models of the middle module.

What could be the function of this middle module? As this region is poorly predicted, and no structural elements were found to resemble the folds of known proteins (data not shown), we present the hypothesis that this part of the protein functions as a linker domain. This is supported by the sequence dissimilarity in this region of the protein (Figure 2). In addition, the previously defined direct-repeat within *At*TOC1 (position 275–369) is not present in orthologous TOC1 proteins. Thus, amino-acid composition of module II appears to be under rapid divergence. We note that a linker is a known feature in separating protein modules, as for example, this is seen in cullin [55] and calmodulin [56]. In each case, linker spacing is critical [57,58]. The sequence degeneration of a putative linker within TOC1 might imply that the PRR polypeptides have dissimilar folds in their middle third. It is also plausible that module II is a native unfolded domain. Perhaps protein length here is more important than a particular structure or amino acid composition.

#### Models of module III

**Figure 6:**
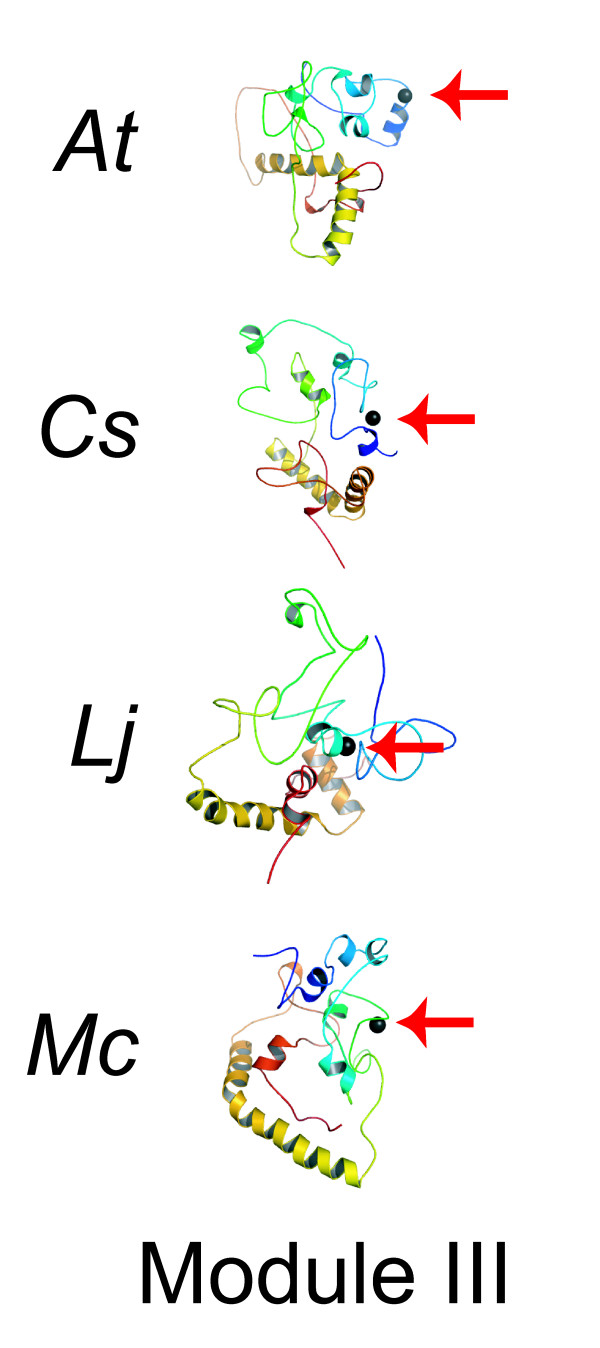
**Models of module III in predictive complex with calcium**. Structural models of module III. The colors from blue to red represent sequence length from the amino- to carboxy-terminal direction. Note that alpha-helical clusters in the carboxy terminus center these structures, and that a calcium ion can be fit into all four structures in an amino-terminal position within all structures. The red arrow points to the fit calcium, which is colored as a gray sphere.

Our final structural efforts targeted the carboxy-termini of the four described TOC1 proteins (Figure 6, Table 1). Unlike module II, each of these was predicted to generate a fold family. All four structures contain two alpha-helices towards the extreme terminus of the protein. This serves to center alignments and represents the CCT sub-domain. This CCT was always found to consist of a small alpha-helical interphase, and in all cases this predicted fold was similar (Figure 6). The overall folding of these structures was found to be predominantly alpha-helical with inter bundle-to-bundle interactions and folded substructures that lack prolonged secondary structure (Figure 6). We further note that module III of TOC1 contains a primary amino-acid composition that does not lend to a detectable primary architecture of known factors. Given the relatedness of the four module III structures, we conclude that the predicted structures could contain structural elements that resemble the true fold.

The presented fold of module III implicates the carboxy terminus of TOC1 in metal binding and also associations to DNA-binding proteins (see below). One interesting feature of the four carboxy-terminal modules is that in structural searches against the three-dimensional folds we generated, each of these four TOC1 modules was found to be in a fold most similar to that present in various metal-binding proteins. Interestingly, the primary amino-acid composition of these domains is unlike that of other metal-binding domains, such as an EF-hand [59]. As the primary and secondary structures of the terminal domain of TOC1 did not detect such relations, we suspect that a structural-folding pattern was required to detect structural elements that relate to biochemical function.

Each TOC1 module III might be related to a metal-binding protein. By SSM searches, we found that the *At*TOC1 structure was most related to calmodulin-sensitive adenylate cyclase (a protein known to be regulated by calcium) [60]; *Cs*TOC1 was most related to calmodulin (a known calcium-binding protein) [61,62]; *Lt*TOC1 was also most related to calmodulin; and *Mc*TOC1 was most related to the zinc-bound form of cell filamentation protein (Structure 2f6s in The Protein Data Bank). Based on the obvious implication that module III could participate in Ca^2+ ^binding, we tried to detect such a binding pocket by a computational approach. Here, we were successful in our ability to fit each of these structures with a bound calcium ion using the GG computational approach [33]. In each case, we could detect that the amino-terminal region of module III harbors a site that could accept the placement of a calcium ion (Figure 6). Note that this is distant from the CCT domain in each case (Figure 6). We thus propose that the third module of TOC1 can be implicated in aspects of metal signaling. This computational finding provides a testable hypothesis for the future.

**Figure 7:**
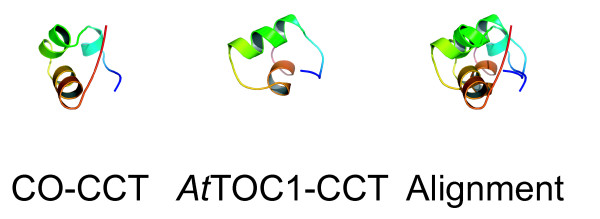
**Comparison of CCT sub-module structures**. From left to right, the predicted structures of the CCT sub-module of CO and *At*TOC1, and their alignment match when aligned. The colors from blue to red represent sequence length from the amino- to carboxy-terminal direction.

We found that the CCT domain within this third of TOC1 was predicted to fold in a similar manner as the CCT domain from CONSTANS (CO) (Figure 7) [63]. As CO is a *bona fide *interactor to HEME ACTIVATOR PROTEIN (HAP) transcription factors [63], it is intriguing that TOC1 could also associate with this class of DNA-binding factors. Two mutant alleles map to the CCT subdomain of module III, and we can thus view the location of these changes. The *toc1-1 *and *toc1-3 *mutations (A562V) both map to an alpha-helical fold within the CCT subdomain, and we note that this Ala residue is conserved in all sequences. The A562V mutation could affect the ability of the CCT to fold into a helix. This would impair its ability to bind target proteins, such as HAP factors. If the hypothesis that the CCT subdomain of TOC1 is a binding interface of HAP factors were true, this would directly implicate TOC1 as a co-regulator of transcription. As *TOC1 *genetically functions to promote *CCA1 *and *LHY *transcription [10,15-18,24], it is an exciting hypothesis that TOC1 functions as a transcriptional co-activator in a multi-protein complex on promoters of clock-regulated genes.

What could be the function of module III in TOC1? It is intriguing that the concentration of cytosolic Ca^2+ ^oscillates with an evening peak close to the time that TOC1 is most abundant [26,29]. cAMPR drives both the circadian oscillations of cytosolic calcium and the rhythmic expression of many clock genes, however not *TOC1 *[2]. It might be that Ca^2+ ^interacts with TOC1 posttranslationally, an idea that is consistent with the fact that calcium rhythms are unaffected in the *toc1-1 *mutant [27]. This calcium interaction would drive the ability of TOC1 protein to regulate its targets. One could thus hypothesize TOC1 to be a component of decoding the Ca^2+ ^signal. If true, TOC1 could generate this function by direct interaction with Ca^2+^. A direct test of Ca^2+^-binding to TOC1 seems a plausible experiment to implicate this protein as a sensor for the circadian levels of Ca^2+^. From there, it would be of interest to test TOC1 binding to HAP factors, and test the role of Ca^2+ ^(or another metal) in supporting or attenuating this binding.

### General considerations of the models and implications of a unified TOC1

How likely are the TOC1 models we present to be correct? This is difficult to assess. In fact, the community standard to answer this question requires the actual structure to be determined [64]. In the absence of an experimentally derived TOC1 structure, we believe that modeling could be useful for predictive biochemistry and to direct further experimentation. We also note that in various benchmarks, ROSETTA correctly predicted protein structures approximately half of the time [36]. We thus conclude that aspects of the model presented here are likely to have useful structural information, but that major structural features could be flawed. Certainly, minor features of the models, such as side-chain directionality, are unlikely to be correct.

**Figure 8:**
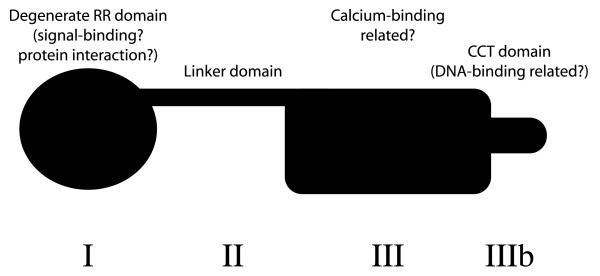
**Schematic representation of a TOC1 structural model**. I PRR domain – this resembles *bona fide *response regulators. II Linker domain – a putative bridge between modules I and III. III Calcium-binding domain – a potential sensor for a metal. IIIb Protein-binding domain – a potential interaction motif for HAP DNA-binding factors.

An over-riding theme generated from our models is the hypothesis that TOC1 acts as a signal adapter that senses a small ligand (*e.g*. Ca^2+ ^or a redox signal) and that this is part of a transcription complex (Figure 8). This multifaceted hypothesis is intriguing given that the plant clock is modulated by small-molecule signaling [65]. For example, redox levels change in response to light [53,66]. Thus, as predicted by Golden and colleagues, the amino-terminus of TOC1 could be involved in metabolite sensing to mediate entrainment. Also, Ca^2+ ^levels coincide with that of TOC1 [26,29]. The scaffold principles implicated from the amino- and carboxy-modules could support a mechanism for TOC1 as a transcriptional mediator that functions in response to signal integration from distinct signaling pathways. This scaffold hypothesis defines the middle module as a tether that links modules I and III. The high degeneration of amino-acid composition in this middle module would support a spacer function rather than a scaffold or enzymatic activity. What is clear is that a biochemical hypothesis now exists to describe how TOC1 leads to transcriptional induction of *CCA1 *and *LHY*.

## Competing interests

The author(s) declare that they have no competing interests.

## Authors' contributions

EK, HS, MP and SJD performed the work. EK and SJD wrote the paper.

## Supplementary Material

Additional file 1 Structural file. Structure of AtTOC1_dom1 Click here for file

Additional file 2 Structural file. Structure of AtTOC1_dom2 Click here for file

Additional file 3 Structural file. Structure of AtTOC1_dom3 Click here for file

Additional file 4 Structural file. Structure of CsTOC1_dom1 Click here for file

Additional file 5 Structural file. Structure of CsTOC1_dom2 Click here for file

Additional file 6 Structural file. Structure of CsTOC1_dom3 Click here for file

Additional file 7 Structural file. Structure of LjTOC1_dom1 Click here for file

Additional file 8 Structural file. Structure of LjTOC1_dom2 Click here for file

Additional file 9 Structural file. Structure of LjTOC1_dom3 Click here for file

Additional file 10 Structural file. Structure of McTOC1_dom1 Click here for file

Additional file 11 Structural file. Structure of McTOC1_dom2 Click here for file

Additional file 12 Structural file. Structure of McTOC1_dom3 Click here for file
